# Superpixel Random Selection Random Walk Multi-Branch Depthwise Convolutional Neural Network for Hyperspectral Image Classification

**DOI:** 10.3390/s26113558

**Published:** 2026-06-03

**Authors:** Kai Zhang, Xinwei Jiang, Zhihua Cai

**Affiliations:** School of Computer Science, China University of Geosciences, Wuhan 430074, China; cug_zk@cug.edu.cn (K.Z.); zhcai@cug.edu.cn (Z.C.)

**Keywords:** CNN, convolutional neural network, deep learning, HSI, hyperspectral image classification

## Abstract

Convolutional neural networks (CNNs) and training-free CNN variants have been successfully applied to hyperspectral image (HSI) processing and analysis. Training-free CNNs have shown promising feature extraction performance, which could effectively address the issue of typical CNNs being highly parameterized; however, inevitable noise and redundancy in the randomly selected training-free convolutional kernels often leads to unsatisfactory performance. To address this issue, we propose Superpixel Random Selection Random Walk Multi-Branch Depthwise Convolutional Neural Network (SRSRWMD-CNN). Specifically, we propose a novel training-free convolutional neural network characterized by inter-layer multi-scale integration and intra-layer grouping. Various superpixels groups are first generated through multi-scale superpixel segmentation algorithms, then the predetermined number of superpixels are randomly sampled from these groups to serve as training-free convolution kernels. This mechanism enables adaptive computation of HSI feature maps without costly model training in the feature extraction stage, allowing the network to effectively capture a multi-scale spectral–spatial feature representation. Additionally, we propose a multi-branch depthwise convolution strategy that mitigates feature learning errors while significantly enhancing feature representation capabilities. A random walk strategy is employed to expand the receptive field and enhance the robustness of the training-free convolution kernels. Finally, the multi-scale spectral–spatial features are concatenated with the multiple convolutional stages to fuse salient shallow and deep features for accurate HSI classification. Extensive experiments demonstrate that the proposed method achieves superior performance compared to state-of-the-art algorithms.

## 1. Introduction

Hyperspectral imaging (HSI) is a powerful technology capable of simultaneously acquiring spatial and spectral information of ground-truth targets [1,2,3,4]. HSI has been successfully applied in numerous tasks, including forest monitoring, urban mapping, climate prediction, and marine exploration [5,6,7]. Hyperspectral image classification (HSIC) is a critical goal of HSI analysis for real-world applications. It has emerged as an active research topic in remote sensing, with the primary objective of assigning a precise ground-truth category to each pixel. HSI data typically consist of hundreds of contiguous spectral bands with complex spatial structures and rich spectral signatures [8]. Pixel-wise annotation of HSI data is labor-intensive, meaning that labeled data often remain limited in practical scenarios. Consequently, achieving accurate HSI classification with limited labeled samples has been challenging, especially when using deep learning-based models.

Over the past few decades, various methods have been developed for HSI classification tasks. Researchers have proposed numerous techniques to exploit the spectral information of hyperspectral images, including random forest models [9], support vector machine (SVM) [10], and K-nearest neighbors (KNN) [11]. However, algorithms that predominantly rely on spectral information can ignore the spatial structural relationships between adjacent HSI pixels, leading to unsatisfactory feature extraction performance. To exploit spatial structural information, researchers have introduced spectral–spatial methods such as Markov random fields (MRF) [12], sparse representation [13], and metric learning [14,15,16]. To deal with the inherent high-dimensional spectral features in HSI, dimensionality reduction techniques such as principal component analysis (PCA) [16] and linear discriminant analysis (LDA) [17] have also been introduced. For example, spectral–spatial HSI classification with parallel implementation based on spatially adaptive MRF was proposed in [12]. This method utilizes a sparse multinomial logistic regression classifier spectrally, after whichspatial information is characterized by modeling the potential function related to weighted MRFs as a spatially adaptive vector total variation function. Chen et al. [13] introduced a novel sparsity-based HSI classification algorithm that incorporates contextual information into the sparsity formulation via two distinct approaches: the first reconstructs the spectral characteristics of the target pixel and its four spatial neighbors, while the second weights the pixels within a small neighborhood area using a set of varying coefficients. However, these traditional methods rely predominantly on feature engineering techniques and lack the capacity to perceive the complex semantic features embedded into high-dimensional HSI data.

In recent years, deep learning-based methods have been widely adopted in hyperspectral image analysis [18]. To ensure consistency between feature extraction and classification, Chen et al. [19] proposed a framework that integrates a deep belief network for hierarchical and semantic spectral–spatial feature extraction with a logistic regression classifier. Liu et al. [20] proposed a sparse-spectral joint stacked autoencoder, integrating spectral features and spatial contextual information of hyperspectral images. Benefiting from the translation invariance and weight sharing of convolution operations, CNNs [21] can effectively capture spatial and spectral features of hyperspectral images. Existing CNN models can be roughly categorized into 1D, 2D, and 3D architectures. For example, Hu et al. [21] applied a 1D-CNN to extract spectral HSI information, achieving higher accuracy than typical shallow models such as SVM-based classifiers. Haut et al. [22] employed a 2D-CNN to perform convolution exclusively across the spatial HSI dimensions to extract the spatial context. Moreover, Hamida et al. [23] utilized a 3D-CNN to perform convolutions simultaneously across both the spectral and spatial dimensions. The evolution from 1D- and 2D-CNNs to 3D-CNNs reflects a progressive deepening in the exploration of spatial–spectral synergies for HSI data analysis.

To efficiently extract spectral and spatial features of HSI, advanced spatial–spectral feature learning models have been proposed recently. For example, Zhong et al. [24] introduced an end-to-end spectral–spatial residual model that leverages residual connections to alleviate the accuracy degradation typically observed in very deep networks. However, 2D-CNNs struggle to extract discriminative features along the spectral dimension, while 3D-CNNs suffer from high computational complexity. To overcome these limitations, Roy et al. [25] proposed a hybrid spectral convolutional model (HybridSN). Their approach first utilizes 3D-CNNs to learn joint spatial–spectral representations, followed by 2D-CNNs to extract more abstract spatial features. Cao et al. [26] proposed a novel framework combining active learning with CNNs to enhance classification performance while minimizing annotation costs. This framework employs a CNN to extract spatial–spectral discriminative features from HSI data and predicts candidate pixel labels, followed by selecting the most informative samples for annotation via active learning. To further extract spatial information from hyperspectral images, Jia et al. [27] combined superpixels, shifting the feature extraction scope from regular images to more realistic irregular images. They achieved structure-adaptive convolution, extracted deep spectral, spatial, and geometric features, then further aggregated information within irregular boundaries. Zhu et al. [28] applied deformable convolutions for HSI classification by utilizing deformable sampling points to adaptively sample the adjacent structural information of each pixel, which effectively captures complex intra-pixel spatial structures in a way that strengthens the model’s spatial expression capability. Zhao et al. [29] proposed a superpixel-guided deformable convolutional network that utilizes the prior structural constraints of superpixel segmentation to constrain the offset learning of deformable convolution, ensuring that the convolution kernel shape aligns with the true boundary contours of land cover. This significantly enhances the accuracy of boundary localization; however, standard deformable convolutions have fixed offsets after training, making it difficult for the model to adapt to unseen deformation patterns in test samples. To address this limitation, MRCAGCFN [30] introduces a random perturbation mechanism into the deformable convolution kernel, breaking through the constraint of fixed shape and achieving adaptive robustness to diverse scenes.

Despite the significant advantages of deep learning-based models in HSI classification, model training remains heavily reliant on large-scale labeled training data, severely restricting the practical application in small labeled sample scenarios [31]. Similar challenges have also been discussed in the non-hyperspectral image processing community. In addition to unsupervised learning strategies, few-shot learning, and data augmentation techniques, training-free deep learning models have also been developed. In these models, numerous parameters can be picked randomly instead of relying on computation-intensive training processes. For example, PCANet [32] is a streamlined deep learning network constructed using cascaded PCA, binary hashing, and block histograms. Although its core PCA filters are derived from data statistical characteristics, they do not require complex iterative optimization. In the HSI domain, scholars have recently proposed a series of CNN models based on random projection theory. These models mitigate reliance on parameter optimization through innovative feature extraction mechanisms. Notably, Xu et al. [33] introduced a feature extraction framework called Random Patch Network (RPNet) based on random patch sampling. This method utilizes hierarchical random sampling of image patches as fixed convolution kernels and employs a cascaded convolution strategy for multi-scale feature fusion, then constructs a classification feature set via concatenation. In this way, it completely avoids the traditional backpropagation-based parameter optimization approach. To further enhance feature expression, local a binary pattern operator was introduced into this framework in [34], significantly strengthening spatial context representation by fusing spatial–spectral joint features. Uchaev et al. [35] innovatively combined random patch networks with recursive filtering, effectively addressing the issues of high complexity and insufficient accuracy in traditional deep learning models for small-sample hyperspectral image classification. Chen et al. [36] proposed the MS-RPNet framework, which integrates S^3^-PCA, 2D-SSA, and a random patch network. This framework stacks multi-scale spatial information through a cascaded structure, then utilizes an SVM to complete the hyperspectral image classification task. Regarding feature optimization, Dundar et al. [37] innovatively applied a guided filtering algorithm to process convolutional features, effectively preserving high-frequency details (e.g., boundaries) and ensuring superior geometric preservation in edge regions. To mitigate intra-class variances during multi-scale feature extraction, Chen et al. [38] proposed a supervector matrix correction algorithm that establishes a multi-scale sub-region association mechanism. A Gaussian smoothing prior is introduced to enhance local consistency in the feature space while maintaining the discriminative power of random patch convolutional features. Although training-free methods based on random patches can effectively alleviate the data-hungry nature of deep networks, the inherent randomness of the sampling mechanism can also introduce noise, leading to inefficient feature representation. To alleviate this problem, Xing et al. proposed Deep Network With Irregular Convolutional Kernels and Self-Expressive Property (DIKS) [39], which merges superpixel segmentation with random sampling strategies. By constraining the patch sampling mechanism within the superpixel boundaries, this approach efficiently aligns the convolution kernels with the spatial distributions of object categories, resulting in significantly improved inter-class separability.

However, training-free convolutional networks such as RPNet and DIKS perform convolutions using features with fixed shapes and receptive fields, making it difficult to adapt to the morphological diversity of targets within hyperspectral images. This inflexibility leads to an inadequate capture of multi-scale features, negatively impacting the accuracy of the HSI classification. In addition, the convolution kernels generated from such fixed features exhibit limited generalization in the feature space compared to convolutional kernels optimized by deep learning models via backpropagation. These suboptimal convolutional kernels can trigger error accumulation during multidimensional feature fusion, ultimately constraining the model’s feature learning performance in complex scenarios. Importantly, utilizing all superpixels directly as convolutional kernels tends to introduce noise. Superpixel segmentation is often disrupted by irregular image textures and illumination changes. Consequently, misclassified or abnormally clustered superpixels that participate in convolution calculations can mislead the feature extraction network, induce overfitting, reduce generalization, and easily cause classification errors.

To address the aforementioned challenges, this paper proposes a novel HSI classification method named Superpixel Random Selection Random Walk Multi-Branch Depthwise Convolutional Neural Network (SRSRWMD-CNN). Specifically, we propose a multi-scale network architecture characterized by inter-layer multi-scale integration and intra-layer grouping. First, various superpixels groups are generated through multi-scale superpixel segmentation algorithms; then, a predetermined number of superpixels is randomly sampled from these groups to serve as a training-free convolution kernel. This mechanism includes inter-layer multi-scale and intra-layer grouping modules that capture multi-scale spatial–spectral features in HSI data, corresponding to different scales of ground-truth objects with varying sizes and fine-grained details. Next, a multi-branch depthwise convolution method is introduced to perform deep convolution with multiple training-free convolutional kernels. This not only mitigates the learned feature error but also significantly enhances the feature representation ability. In addition, a superpixel random walk kernel generation method is proposed to enhance the robustness of the convolutional kernels, expand the receptive field, and acquire features from a broader spatial perspective. Finally, the multi-scale spectral–spatial features are concatenated with the multiple convolutional stages to fuse the shallow and deep features, leading to salient spatial–spectral features for accurate HSI classification.

The remainder of this paper is organized as follows: Section 2 briefly reviews related works; the proposed method is introduced in Section 3, followed by experimental results and analysis in Section 4; finally, our conclusions are drawn in Section 5.

## 2. Related Work

### 2.1. Random Walks

Random walks play a pivotal role in machine learning, particularly within the domain of HSI classification. The theoretical foundations span probability theory, combinatorial mathematics, and stochastic processes. Classical random walk models focus on properties such as stationary distributions and first passage times, providing robust theoretical support for applied research.

The specific implementation steps are as follows: assume there is a finite set of points $X=\{x_{1},x_{2},\ldots ,x_{N}\}$ in space; starting from an initial point in space, at each step the point moves randomly to another point in space based solely on the current point, following a fixed state transition probability regardless of the historical path. The overall behavior is characterized by random diffusion-like movement of the point set in space. Let $\pi_{k}$ denote the probability distribution of the point set in space at *k* step and let *P* denote the transition matrix in space; then, the process of random walk can be represented as

(1) $$\pi_{k+1}=\pi_{k}P.$$

After *k* steps, the probability distribution on the point set is

(2) $$\pi_{k}=\pi_{0}P^{k},$$

where $\pi_{0}$ is the initial point distribution.

In HSI classification, random walks leverage both spectral and spatial information: by initially constructing a relational graph among image pixels, the algorithm performs random walks on the graph to propagate category information from labeled pixels to unlabeled ones. This mechanism can effectively address the scarcity of labeled samples in hyperspectral datasets, leading to improved classification accuracy. Moreover, by integrating intrinsic properties of hyperspectral images such as spectral curve similarities and spatial neighborhood dependencies, the performance of the random walk model can be significantly enhanced.

Additional variants and extensions of random walks have introduced novel paradigms to HSI classification. For example, weighted random walks can propagate information more accurately across complex HSI structures by incorporating weights derived from spectral similarity and spatial distance. When accounting for the high-dimensional nature of hyperspectral data, multidimensional random walks can systematically analyze the correlations and interactions across varying bands, thereby mining deeper discriminative information. These advances offer effective strategies for HSI classification, bringing improved predictive performance and more detailed exploitation of the underlying data structure of HSI.

### 2.2. Training-Free Convolutional Networks

Various training-free convolutional networks have been proposed, such as PCANet, RPNet, and DIKS, etc., seeking to effectively address the issue of high parameterization in typical CNNs. Such approaches have shown promising performance in extracting HSI features. PCANet is a streamlined deep learning architecture built on cascaded PCA, binary hashing, and block histograms. The core PCA filters of the network are computed by deriving the eigenvectors of the covariance matrix from image patches extracted from the training set. This process relies strictly on the statistical properties of the data and circumvents complex iterative optimization. The primary convolution process involves cropping the image into patches across each dimension and stacking them to form $X\in R^{ww\times Nmn}$, where *w* denotes the patch size, *N* represents the input channel dimension, and mn indicates the total number of patches (the product of horizontal and vertical patch counts). Subsequently, PCA is applied to reduce the dimensionality to *L*, generating the convolution kernels $W\in R^{w\times w\times L}$:

(3) $$W_{l}={\mathrm{mat}}_{w,w}\left(q_{l}\left(XX^{T}\right)\right),\;l=1,2,\ldots ,L.$$

Following this, a convolution operation is performed to produce the output tensor $O\in R^{w\times w\times LN}$:

(4) $$O=I_{i}*W,\;i=1,2,\ldots ,N.$$

A notable variant is RandNet, which replaces the PCA filters with randomly generated filters to entirely eliminate the training phase. Another variant, LDANet, utilizes filters learned from linear discriminant analysis (LDA).

In the field of HSI classification, Xu et al. [33] proposed the Random Patch Network (RPNet). Inspired by random projection theory, this method employs randomly extracted image patches as convolution kernels without requiring any training. Random projection theory effectively maps high-dimensional data to a lower-dimensional random subspace while preserving both the original structural integrity and discriminative capability of the data. The convolution process primarily entails randomly selecting *k* patches from the input to serve as convolution kernels $P_{1},P_{2},\ldots ,P_{k}\in R^{w\times w\times q}$. The convolution operation is then executed to yield the output feature map $I\in R^{m\times n\times k}$:

(5) $$I_{i}=\sum_{j=1}^{q}X^{\left(j\right)}*P_{i}^{\left(j\right)},\;i=1,2,\ldots ,k.$$

To capture the nonlinear relationships present in the input HSI, rectified linear unit (ReLU) is subsequently applied as the activation function for each convolutional layer:

(6) $$\sigma \left(I_{j}^{p}\right)=\max\left(0,I_{j}^{p}-{\bar{I}}_{j}\right).$$

RPNet integrates both shallow and deep convolutional features during extraction, facilitating multi-scale analysis and demonstrating robust adaptability to targets of varying sizes. Compared to traditional deep learning paradigms, RPNet boasts a simpler architecture and completely eliminates the dependence on large-scale training samples for feature extraction.

Similarly, Deep Network With Irregular Convolutional Kernels and Self-Expressive Property (DIKS) [39] leverages irregular convolutional kernels and self-expressive properties to elevate classification performance. DIKS generates a series of irregular convolution kernels via PCA combined with superpixel segmentation, enabling these kernels to adaptively delineate the distinct features of different object categories. The design philosophy of DIKS also draws heavily from random projection theory, capturing complex structural semantics within HSI through these irregular kernels. The convolution mechanism of DIKS parallels that of RPNet, with the critical distinction being that the standard kernels are replaced by zero-padded superpixels. Furthermore, DIKS fuses shallow and deep features, then clusters them using self-expression theory, which magnifies feature discriminability. Similar to RPNet, DIKS does not require extensive training data, and the incorporation of irregular kernels endows it with superior adaptability to complex hyperspectral inputs.

Although promising experimental results have been demonstrated, existing training-free convolutional networks such as RPNet and DIKS perform convolutions using features with fixed shapes and receptive fields, making it difficult to adapt to the morphological diversity of targets within hyperspectral images.

## 3. Methodology

This section introduces the proposed SRSRWMD-CNN.

**Figure 1:**
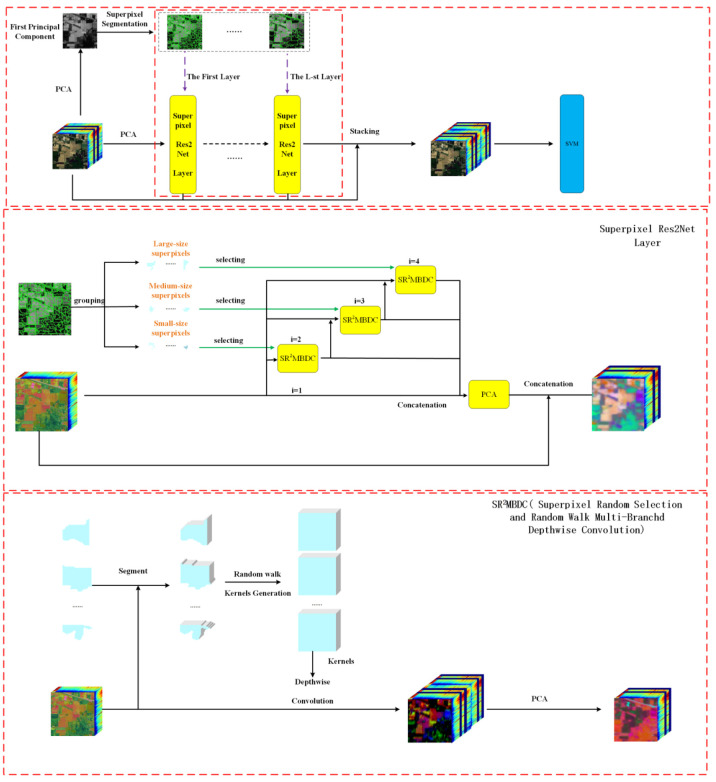
SRSRWMD-CNN

As can be seen from the flowchart in Figure 1, we first use the ERS algorithm to segment the image in order to obtain various superpixels at different scales. Then, we feed the PCA preprocessed data into stackable superpixel Res2Net layers, where each layer corresponds to a superpixel segmentation scale. In each layer, we group and select superpixels based on different scales. We then conduct superpixel random selection and random walk multi-branch depthwise convolution ($SR^{2}MBDC$) in the proposed superpixel Res2Net module, which aims to obtain training-free convolution kernels through the proposed superpixel random walk kernel generation and extract features through random selection multi-branch depthwise convolution. Finally, the extracted features from each layer are stacked with the original features to form the multi-scale spatial–spectral features which are fed into SVM for classification.

Let $H\in R^{h\times w\times b}$ denotes the original hyperspectral image data, where *h*, *w*, and *b* represent the height, width, and number of spectral bands, respectively. Initially, typical PCA is employed to reduce the spectral dimension to 1 and *Q* dimensions, yielding the first principal component $FC\in R^{h\times w}$ and the initial input tensor $I\in R^{h\times w\times Q}$. Because the first principal component encapsulates the most salient spatial information of the HSI, it serves as the base image for entropy rate superpixel (ERS) segmentation, generating superpixel segmentation maps $SM_{l}\;\left(1\leq l\leq L\right)$, where *L* denotes the total number of convolutional layers. The number of superpixels varies across layers, with the scale of each layer denoted as $P_{l}$:

(7) $$P_{l}=P_{0}\times \left(L-l+1\right).$$

### 3.1. Superpixel Res2Net Layer

In each superpixel Res2Net layer, the superpixel segmentation scale is $P_{l}$ and each layer corresponds to a superpixel segmentation map $SM_{l}$. Each superpixel Res2Net layer can obtain the output $O_{l}\in R^{h\times w\times Q}$ from each layer. The final extracted feature is $O=[H,O_{1},\ldots ,O_{L}]$, which is fed into the SVM training classifier. In a superpixel segmentation map $SM_{l}$, we define $d_{l}^{j}$ to be the j-th superpixel size in the scale

(8) $$d_{l}^{j}=\max\left(h_{\max,l}^{j}-h_{\min,l}^{j},w_{\max,l}^{j}-w_{\min,l}^{j}\right),$$

where $h_{\max}$, $h_{\min}$, $w_{\max}$, and $w_{\min}$ represent the maximum and minimum spatial coordinates of all pixels encompassed by the specific superpixel. The map $SM_{l}$ is then partitioned into three equally sized groups, denoted as $G_{l}^{1},G_{l}^{2},G_{l}^{3}$, which correspond to small-sized, medium-sized, and large-sized superpixels, respectively.

**Figure 2:**
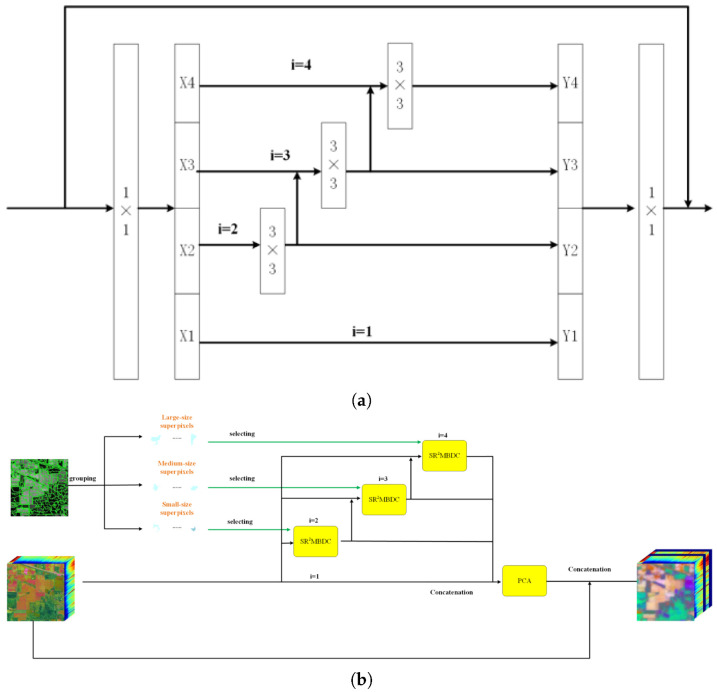
Res2Net module (**a**) and superpixel Res2Net layer (**b**).

As shown in the Figure 2, we adopt a similar network structure to the Res2Net module, with the difference being that we use a training-free superpixel random selection and random walk multi-branch depthwise convolution operation plus PCA to replace the typical convolution operation in Res2Net. In order to adapt to the training-free convolution operation, we transform the channel-wise processing of Res2Net into multi-branch full channel processing and change the residual connection from addition to cascade for more effective feature extraction.

Let $K_{i}\left(\cdot \right)$ denote the superpixel random selection and random walk multi-branch depthwise convolution operation, where i represents the different branches of the structure, $y_{i}$ represents the output of $K_{i}\left(\cdot \right)$, and *x* represents the input of the convolution layer. Consequently, $y_{i}$ is formulated as shown in the equation below.

(9) $$y_{i}=\left\{\begin{array}{cc} x & i=1;\\ K_{i}\left(x,G_{l}^{1}\right) & i=2;\\ K_{i}\left(cat\left(x,y_{i-1}\right),G_{l}^{i-1}\right) & 2<i\leq 4. \end{array}\right.$$

This multi-scale approach is highly effective for capturing rich and valuable spatial–spectral features that can facilitate highly accurate HSI classification. To optimally fuse information across varying scales, we concatenate all groups along the channel dimension such that $y=[y_{1},y_{2},y_{3},y_{4}]$, where […] denotes the concatenation operation. Finally, PCA is applied to extract the top *Q*-dimensional features, yielding the final output feature map in each layer $O_{l}\in R^{h\times w\times Q}$ and the input of the next layer $I_{l+1}=\left[I_{l},O_{l}\right]$.

### 3.2. Random Selection Multi-Branch Depthwise Convolution

**Figure 3:**
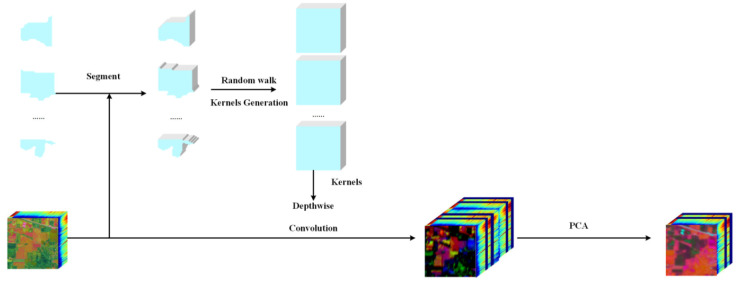
Random selection-based multi-branch depthwise convolution.

To minimize convolution errors and extract optimally representative spatial–spectral features, as shown in Figure 3, we propose random selection-based multi-branch depthwise convolution. Let $I_{l}^{i}\in R^{h\times w\times Q}$ denote the input to $K_{i}\left(\cdot \right)$ at the *l*-th layer. Superpixels are randomly sampled from the corresponding superpixel group $G_{l}^{i}$ to form a subset $g_{l}^{i}=\left\{sp_{l,1}^{i},sp_{l,2}^{i},\ldots ,sp_{l,n}^{i}\right\}$, where *n* is the number of randomly selected superpixels. Through the superpixel random walk kernel generation process, the convolution kernels $W\in R^{D_{l}^{i}\times D_{l}^{i}\times Q\times n}$ are derived from $g_{l}^{i}$, where $D_{l}^{i}$ represents the maximum superpixel size $d_{l}^{j}$ within group $G_{l}^{i}$. We mathematically formulate the convolution layer as follows:

(10) $$Z=\left\{Z_{1}^{1},Z_{1}^{2},\ldots ,Z_{1}^{Q},Z_{2}^{1},Z_{2}^{2},\ldots ,Z_{n}^{Q}\right\},$$

(11) $$Z_{j}^{q}=w_{j}^{q}*i^{q},\;1\leq q\leq Q,\;1\leq j\leq n,$$

where $Z\in R^{h\times w\times nQ}$ represents the output feature map after convolution, while $w_{j}^{q}\in R^{D_{l}^{i}\times D_{l}^{i}}$ and $i^{q}\in R^{h\times w}$ correspond to the *q*-th channel of the weights and input, respectively. To capture the inherent nonlinearities within HSI data, the ReLU activation function is applied following each convolution operation. Similar to typical training-free convolution neural networks such as RPNet and DIKS, we use the typical ReLu in this paper, as it provides nonlinear feature processing while enhancing sparsity, filtering out invalid backgrounds and noise, and suppressing random interference caused by training-free convolution kernels. Finally, it provides the proposed training-free convolution neural network with significant feature expression ability. The ReLu activation function used in the paper can be formalized by

(12) $$\sigma \left(Z_{j}^{q}\right)=\max\left(0,Z_{j}^{q}-\bar{Z}\right),$$

where $\bar{Z}\in R^{h\times w}$ denotes the mean matrix computed across *Z*. Utilizing multi-branch depthwise convolution together with concatenation of each branch triggers rapid dimensionality expansion, which inevitably introduces data redundancy. Therefore, a final PCA reduction is executed to extract the most discriminative *Q*-dimensional features, producing the final output tensor $y_{i}\in R^{h\times w\times Q}$.

### 3.3. Superpixel Random Walk Kernel Generation

**Figure 4:**
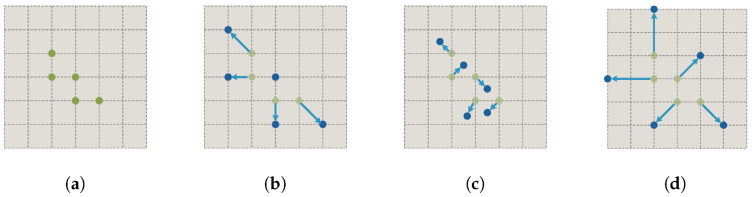
Four distinct convolutional kernel paradigms: (**a**) vanilla superpixel convolutional kernel, (**b**) dilated superpixel convolutional kernel, (**c**) random shape superpixel convolutional kernel, and (**d**) random walk superpixel convolutional kernel.

To enhance the robustness of the convolution operation and expand the effective receptive field, we introduce superpixel random walk convolution kernel generation. As shown in Figure 4, the proposed superpixel random walk kernel generation approach ensures a significantly expanded field of view while exhibiting structural flexibility similar to that of superpixel random shape convolution.

We directly utilize the superpixels acquired from $g_{l}^{i}$ to segment $I_{l}^{i}$ into numerous irregular 3D patches. To ensure mathematical compatibility with standard convolution routines, we initially apply zero-padding to expand these irregular shapes into uniform square kernels. To standardize the dimensions, the kernel size is fixed at $D_{l}^{i}$, resulting in padded patches represented as $w_{p}\in R^{D_{l}^{i}\times D_{l}^{i}\times Q\times n}$. We assume that each spatial coordinate within the kernel is connected to its immediate neighbors in a checkerboard pattern (including self-connections).

In traditional hyperspectral image processing approaches such as [40], the random walk method is based on the similarity between points and the surrounding areas, which is then used to generate random walk weights. The higher the similarity, the greater the weight of the random walk. Instead, we consider superpixels as convolution kernels in order to expand the receptive field of superpixels and make their shape not fixed, which improves the robustness. Our approach performs random walks in a fixed space filled with domain information, meaning that it is difficult to define which results are considered most optimal when designing analytical solutions. Therefore, the formulation of Brownian motion is introduced to obtain weights; we then reverse the weight definition in Equation (13) to increase the likelihood of outward expansion, while the boundaries are set to prevent infinite expansion.

Consequently, an adjacency matrix $A\in R^{\left(D_{l}^{i}\times D_{l}^{i}\right)\times \left(D_{l}^{i}\times D_{l}^{i}\right)}$ is constructed:

(13) $$A_{i,j}=\left\{\begin{array}{cc} 1, & \mathrm{if}\;\left|x_{i}-x_{j}\right|+\left|y_{i}-y_{j}\right|\leq 1,\\ 0, & \mathrm{otherwise}. \end{array}\right.$$

To effectively expand the receptive field and encourage the outward diffusion of superpixels away from the geometric center, we derive the random walk transition matrix $T\in R^{\left(D_{l}^{i}\times D_{l}^{i}\right)\times \left(D_{l}^{i}\times D_{l}^{i}\right)}$:

(14) $$T_{i,j}=\left\{\begin{array}{cc} \exp\left(-\frac{dis_{max}^{2}-dis_{i,j}^{2}}{4t}\right), & \mathrm{if}\;A_{i,j}=1,\\ 0, & \mathrm{otherwise}, \end{array}\right.$$

where $dis_{i,j}$ denotes the Euclidean distance between a given point and the center while $dis_{max}$ represents the maximum possible value of $dis_{i,j}$. To guarantee that the transition probabilities originating from any single point sum to 1, the matrix *T* is strictly normalized.

By performing an exponential operation on the transition matrix, we calculate the final landing probability matrix of all points after *m* steps of random walk, denoted as $P\in R^{\left(D_{l}^{i}\times D_{l}^{i}\right)\times \left(D_{l}^{i}\times D_{l}^{i}\right)}$:

(15) $$P=T^{m}.$$

The superpixels actually perform random walks in an area of size $D_{l}^{i}\times D_{l}^{i}$, and their final resting position is also within this area; thus, the stability of the random walk is ensured and the final state tends to converge smoothly in the boundary region.

Finally, we employ the roulette wheel selection algorithm, in which the initial point sets within each convolution kernel of $w_{p}$ are stochastically mapped to their final coordinates within the $D_{l}^{i}\times D_{l}^{i}$ spatial grid, thereby synthesizing the final convolution kernel *W*.

**Figure 5:**
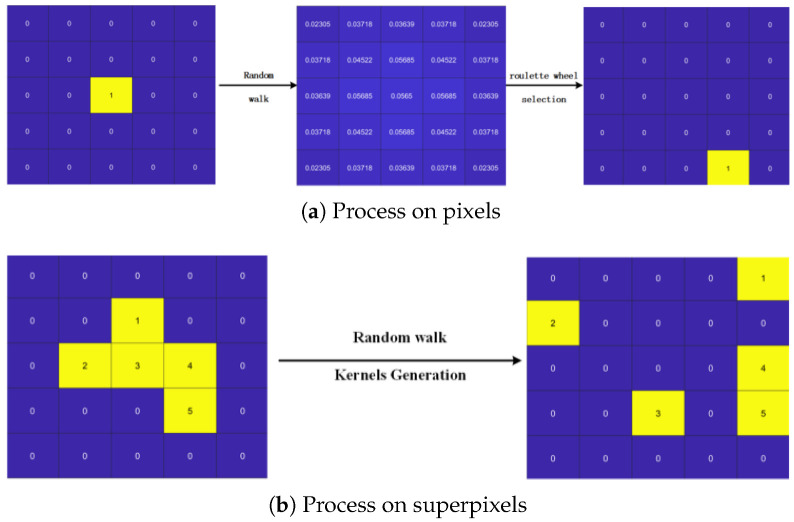
The random walk and roulette wheel selection process.

As shown in Figure 5, taking as an example a scenario where we select a point randomly and then walk five steps in a 5 × 5 area, we obtain the probability of the center point’s final position in the 5 × 5 area through Equation (14) and determine its final position using the roulette wheel selection method. Similarly, by repeating this operation, we can determine the final position of each point in each superpixel after random walks to obtain a new superpixel convolution kernel. It can be clearly seen that the newly obtained superpixel convolution kernel can obtain a larger receptive field compared to existing models and is not fixed in its original shape.

## 4. Experiments

To evaluate the effectiveness of the proposed framework, extensive experiments are conducted on three widely recognized benchmark HSI datasets, comparing classic and state-of-the-art trainable and training-free deep learning models. The code of the paper is available at https://github.com/ZhangKaiCug/SRSRWMD-CNN (accessed on 24 May 2026).

Three HSI datasets are used in the following comparative experiments. The first is the Indian Pines (IP) dataset, which was acquired using the Airborne Visible/Infrared Imaging Spectrometer (AVIRIS) sensor to capture agricultural landscapes in northwestern Indiana, USA. The spectral range spans from 400 nm to 2500 nm (visible to short-wave infrared), originally comprising 224 spectral bands. After discarding bands afflicted by noise and water absorption, 220 functional bands remain for analysis. The dataset features spatial dimensions of 145×145 pixels, with a spatial resolution of 20 m per pixel.

The second is the Pavia University (PU) dataset, which was collected using the Reflective Optics System Imaging Spectrometer (ROSIS) sensor over the urban expanse surrounding the University of Pavia, Italy. This dataset covers a wavelength range from 430 nm to 860 nm, and initially included 115 spectral bands. Following the removal of noisy bands, 103 bands are retained for analysis. The dataset features spatial dimensions of 610×340 pixels, boasting a high spatial resolution of 1.3 m per pixel.

Finally, the Houston 2013 (HOU) dataset was acquired by the ITRES CASI-1500 sensor (Calgary, AB, Canada) in 2013 by surveying the University of Houston campus and its adjacent urban areas in Texas, USA. It provides a spatial resolution of 2.5 m and encompasses 144 spectral bands, covering a wavelength spectrum from 380 nm to 1050 nm.

To quantitatively assess the classification fidelity of the evaluated methods, three standard evaluation metrics are employed: Overall Accuracy (OA), Average Accuracy (AA), and Kappa coefficient (κ). OA denotes the proportion of correctly classified pixels relative to the total number of test pixels, AA represents the mean percentage of correct classifications across all individual categories, and the Kappa coefficient serves as a robust statistical measure that corrects the baseline accuracy by accounting for the probability of chance agreement.

### 4.1. Parameter Selection and Sensitivity Analysis

To comprehensively evaluate the performance of the proposed model, it is significant to first analyze the sensitivity of six primary hyperparameters in our model: the superpixel segmentation scale *P*, number of network layers *L*, feature dimension during convolution *Q*, number of randomly selected convolution kernels *n*, random walk steps *m*, and temperature coefficient *t*. We randomly select five labeled samples from each category to form a training set and repeat this process ten times to obtain the average value of the performance metrics.The specific details are shown in Table 1, Table 2 and Table 3.

First, considering the varying spatial resolutions and dimensions of the datasets, we define distinct search intervals for the optimal superpixel segmentation scale. For the IP dataset, the range was set to [20,520] with an interval of 10. For the PU dataset, the range was [200,1200] with an interval of 20. For the HOU dataset, the interval spanned [5000,15,000] with a step size of 200.

**Figure 6:**
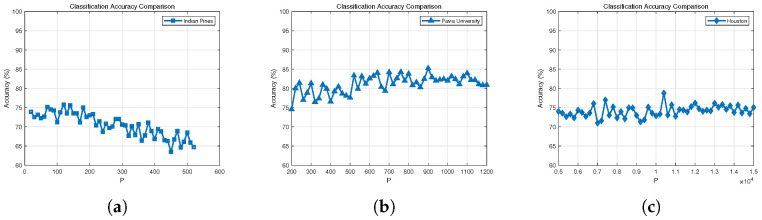
OA results corresponding to different values of the superpixel parameter *P* on the three HSI datasets: (**a**) IP, (**b**) PU, and (**c**) HOU.

As depicted in the associated Figure 6, as the number of superpixels increases, the OA typically follows a parabolic trajectory, initially rising and subsequently declining. A relatively small number of superpixels fails to capture adequate spatial detail, whereas an excessively large *P* introduces fine-grained noise that degrades the quality of the convolutional kernels. Empirical evidence reveals that the optimal values for the three datasets are P=120, 900, and 10,400, respectively.

**Figure 7:**
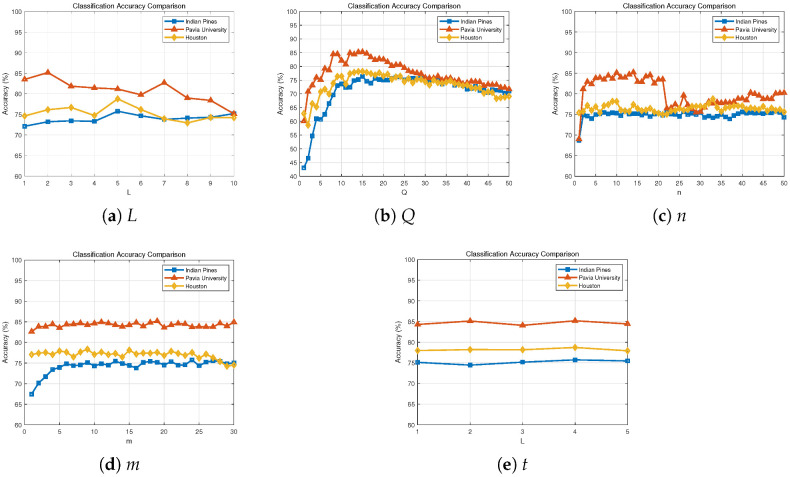
OA results corresponding to different parameters: *L*, *Q*, *n*, *m*, and *t*.

Next, we investigate the optimal the number of network layers *L* within the range [1,10] for all three datasets. As shown in the Figure 7, increasing the layer count similarly leads to an initial surge in OA followed by degradation. While shallow networks lack the capacity to extract high-level abstract features, overly deep networks are susceptible to overfitting and data distortion in few-shot scenarios. Consequently, the optimal layer counts are determined to be L=5, 2, and 5 for the IP, PU, and HOU datasets, respectively.

Next, we examine the optimal feature dimension *Q* within the range [1,50]. as depicted in the associated Figure 7, the analytical results indicate that OA initially improves with higher dimensions before steadily declining. An excessively large feature dimension inevitably triggers the curse of dimensionality issue, in which redundant or noisy information obstructs classification; conversely, a severely restricted dimension truncates critical discriminative information. Interestingly, this hyperparameter demonstrates consistently optimal behavior across all three datasets, stabilizing at Q=15.

Finally, we perform sensitivity analysis on the number of randomly selected convolution kernels *n*, evaluating the values ranging [1,50].

As illustrated in Figure 7, the PU dataset displays a clear inverted-U trend for OA as *n* increases. An overabundance of kernels introduces redundant noise, while an insufficient pool of kernels restricts the model’s feature representation capacity. However, this parabolic trend is less pronounced on the IP and HOU datasets. The IP dataset possesses a relatively coarse spatial structure, rendering the impact of kernel noise less critical; conversely, the highly refined superpixel segmentation in the HOU dataset intrinsically mitigates the adverse effects of redundant kernels. Ultimately, the optimal parameters are set to n=12, 14, and 33 for the three datasets, respectively.

Finally, as illustrated in Figure 7, the experimental results on the IP and PU datasets show that the OA first rises with the increase of *m*, then tends to flatten out; the robustness provided by random walks is also demonstrated. However, on the HOU dataset there is a gentle upward trend followed by a downward trend. Due to the relatively fine segmentation of this dataset, the drawbacks of excessive random walks can become apparent and disrupt the original form. Ultimately, the optimal parameters for the three datasets are set to m=24, 19, and 9, respectively.

As shown in Figure 7, the temperature coefficient *t* has little effect on the experimental results, and the curve tends to be flat. Therefore, we chose the optimal value as t=4.

### 4.2. Ablation Experiments

To validate the efficacy of the distinct modules in our model, extensive ablation studies are conducted on the three datasets. We randomly select five labeled samples from each category to form a training set and repeat this process ten times to obtain the average values of the performance metrics.

As shown in the Table 4, Res2Net acts as the backbone architecture; in addition, “multi-scale” indicates the strategic variation in the numbers of superpixel segmentation scales across layers, “grouping” denotes the intra-layer segregation of superpixels according to scale, “random selection” refers to the stochastic kernel-picking process, “depthwise convolution” confirms the integration of channel-wise decoupled operations, “multi-branch” indicates the concatenation of features derived from parallel depthwise convolution paths, and “superpixel random walk kernel generation” reflects the synthesis of expansive kernels generated via superpixel random walk kernel generation trajectories mapped over the original kernels.

The proposed model achieves exemplary OA scores of 75.78%, 85.17%, and 78.78% on the IP, PU, and HOU datasets, respectively, along with Kappa coefficients of 72.68%, 81.25%, and 77.06%, respectively. Removing the Res2Net backbone induces OA degradations of 1.80%, 5.30%, and 8.59% and Kappa reductions of 1.98%, 6.64%, and 9.29%, respectively, verifying that the Res2Net topology is indispensable for extracting highly detailed features. Eliminating the multi-scale mechanism causes the OA to decline by 1.17%, 1.94%, and 6.27%, respectively, while omitting the grouping module decreases the OA by 0.94%, 6.13%, and 3.37%. These results empirically confirm that the multi-scale grouping architecture is vital for comprehensively perceiving multi-scale target dimensions and fine-grained spatial information.

If the random selection strategy is excluded, the respective OA values drop by 0.94%, 0.09%, and 13.68%, highlighting its crucial role in boosting robustness and suppressing data redundancy and noise. When depthwise convolution is bypassed, severe OA declines of 9.41%, 17.88%, and 5.77% are observed, alongside Kappa drops of 10.62%, 21.71%, and 4.04%, respectively, underscoring the critical importance of depthwise convolutions in mitigating the error accumulation effect during multidimensional feature fusion. Omitting the multi-branch logic reduces OA by 7.07%, 16.13%, and 3.37%, respectively, demonstrating that parallel branching effectively enriches structural representations. Finally, ablating the superpixel random walk kernel generation leads to OA decreases of 8.22%, 3.81%, and 1.02%. This unequivocally illustrates that the random walk-driven kernel synthesis profoundly fortifies convolutional robustness and captures features from a substantially broadened receptive field.

As shown in the Table 5, first, the proposed strategy of superpixel random walk kernel generation is verified by comparing it to superpixel dilated kernel generation and superpixel random shape kernel generation. The empirical data conclusively demonstrate that proposed superpixel random walk kernel generation approach achieves optimal OA and Kappa metrics across all three datasets. This superiority stems from its unique ability to simultaneously foster structural robustness and acquire a vastly expanded spatial receptive field. Interestingly, naive fusion of superpixel dilated kernel generation and superpixel random shape kernel generation failed to yield synergistic gains, with its performance on the PU and HOU datasets even underperforming the single-operation baselines. This compellingly demonstrates that optimally balancing receptive field dilation and morphological robustness requires a sophisticated architectural integration, precisely as orchestrated by the proposed superpixel random walk kernel generation strategy.

### 4.3. Comparison Experiments

For this comparison, we randomly select five labeled samples from each category to form the training set and repeat this process ten times to obtain the average value of the performance metrics.

**Comparative Methods:** To empirically verify the superiority of the proposed framework, we evaluate it against nine state-of-the-art algorithms: SVM, RPNet [33], DIKS [39], GRR [37], M3FUNET [38], CEGCN [41], SACNN [27], SSLSM [42], 3DCT [43], MSFI-CNet [44], and SSFEN [45]. The baseline SVM utilizes the original spectral profiles directly for classification. RPNet employs a CNN-like random patch network to extract deep features, which are subsequently classified by an SVM. DIKS expands upon RPNet by building a network that strictly adopts superpixels as convolutional kernels. GRR pairs guided filtering with random patch networks to jointly compute multiple feature sets for comprehensive final classification. M3FUNET operates via a dual-path architecture: one path corrects supervector matrices to mitigate intra-class variances across multi-scale regions, while the second path incorporates Gaussian smoothing into a random patch network for robust feature extraction. CEGCN leverages CNNs to process small-scale local targets and GCNs to aggregate features from irregularly shaped broader regions. SACNN utilizes CNNs to evaluate superpixels, performing pixel classification driven entirely by superpixel clustering boundaries. SSLSM utilizes a spectral masking technique to self-reconstruct masked bands, enhancing the model’s extraction of global semantic information while minimizing spectral noise. 3DCT bridges local-level CNN extraction with global-level transformer encoding. MSFI-CNet is a dual-branch transformer network that combines sparsification and high-low frequency interaction. SSFEN has designed a spatial spectral enhancement strategy and adaptive decision fusion to enhance the feature discrimination ability from both spatial and spectral perspectives, which is used to fuse all features.

The results for SVM, RPNet, DIKS, GRR, M3FuNet and the proposed model are all based on Ubuntu 22.04.5 LTS, MATLAB2020b, i9-12900K CPU, and 128 GB of RAM; graphics processing unit (GPU) acceleration was not used. The results for CEGCN, SACNN, SSLSM, 3DCT, MSFI-CNet, and SSFEN are all based on Ubuntu 16.04.6 LTS, Pytorch 1.9 framework in PyCharm 2022.1.1, E5-2678 CPU, NVIDIA GeForce RTX 3080 GPU (12 GB) (Nvidia, Santa Clara, CA, USA), and 64 GB of RAM.

**Figure 8:**
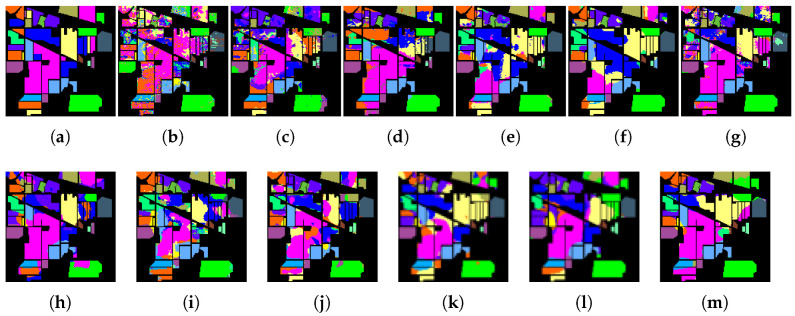
Classification maps generated by different methods on the Indian Pines dataset: (**a**) ground truth, (**b**) SVM, (**c**) RPNet, (**d**) DIKS, (**e**) GRR, (**f**) M3FUNET, (**g**) CEGCN, (**h**) SACNN, (**i**) SSLSM, (**j**) 3DCT, (**k**)MSFI-CNet, (**l**) SSEFN, and (**m**) proposed method.

On the IP dataset, the proposed model demonstrates superior performance across the three crucial metrics of OA, AA, and Kappa compared to all benchmarked algorithms. As shown in the Table 6, the traditional SVM consistently establishes the lowest performance baseline on the IP dataset, followed by RPNet. DIKS and SSLSM demonstrate comparable OA metrics, hovering around 67%. GRR, M3FUNET, and MSFI-CNet, serving as optimized iterations of RPNet, display an accuracy increment of approximately 12% over the base RPNet architecture. The three modern learning networks CEGCN, SACNN, and 3DCT all deliver commendable results, achieving OA scores of 73.58%, 74.91%, and 72.43%, respectively. Nonetheless, the proposed model firmly secures the best overall performance, surpassing the second-best algorithm in OA by 0.87%. As shown in the Figure 8, a visual inspection of the IP classification maps reveals that SVM suffers from pervasive noise and degraded object boundaries. While RPNet and DIKS partially restore regional consistency, they suffer from localized misclassifications and discontinuous segmentations in the diagonal zones. GRR and M3FUNET struggle to accurately trace complex disjointed boundaries and routinely misjudge isolated small-area ground targets. SACNN is penalized by a pronounced “blocking effect” stemming from rigid superpixel voting, which aggressively erases fine-grained targets. Although SSLSM significantly curtails noise, erratic coloring still blemishes the periphery of the *Soybean-mintill* zones. Similarly, while 3DCT refines global structural alignment, anomalous misclassification clusters stubbornly persist within the *Woods* territory. Although MSFI-CNet can capture spectral details to identify unique features, it is not able to effectively model the characteristics of similar crops under different cultivation conditions. SSFEN performs exceptionally well in category recognition for homogeneous and well-sampled land features such as *Hay-winded*, *Grass-tree*, and *Woods*. However, it severely misjudges small sample or high intra-class heterogeneity categories such as *Grass-pasture-mowed*, *Oats*, and *Alfalfa*, reflecting insufficient learning of fine-grained spectral features in the cases of small training samples. In contrast, the proposed classification model is able to track diagonal topographies, eliminate noise within the *Soybean-mintill* regions, and preserve fine-grained ground objects.

**Figure 9:**
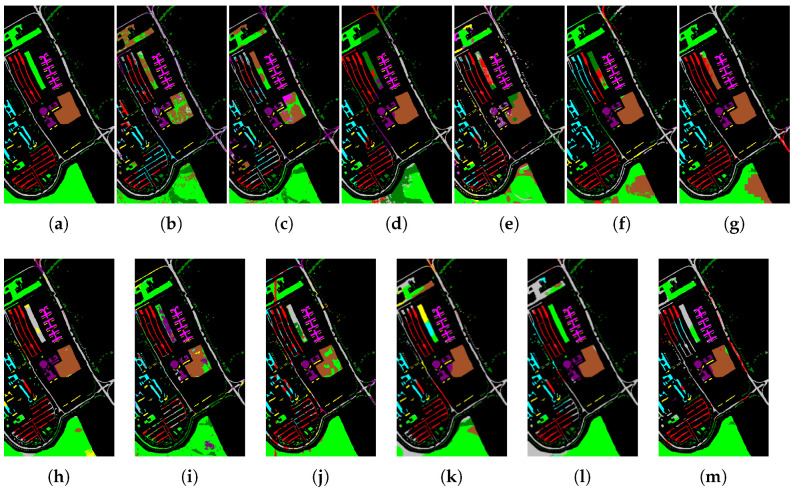
Classificationmaps generated by different methods on the Pavia University dataset: (**a**) ground truth, (**b**) SVM, (**c**) RPNet, (**d**) DIKS, (**e**) GRR, (**f**) M3FUNET, (**g**) CEGCN, (**h**) SACNN, (**i**) SSLSM, (**j**) 3DCT, (**k**) MSFI-CNet, (**l**) SSEFN, and (**m**) proposed method.

The proposed model concurrently sustains superior performance on the PU dataset. Traditional SVM once again yields the lowest accuracy baseline. As shown in the Table 7, RPNet and its derivative variants (DIKS, GRR, M3FUNET) produce OA values between 71% and 74%. SSLSM, 3DCT, and MSFI-CNet output similar OA percentages of approximately 79%, while CEGCN and SACNN record highly competitive values near 84%. Nevertheless, the proposed model dominates the field, outperforming the leading competitor by 0.88% in OA. As shown in the Figure 9, inspecting the associated visual classification maps, SVM is plagued by widespread variegation, rendering the boundaries of the *Asphalt* regions virtually indistinguishable. RPNet and DIKS manifest edge labeling inaccuracies, omitting crucial structural details within the *Asphalt* class and routinely misclassifying constrained spatial zones. Although GRR and M3FUNET successfully mend some discontinuities in tracking the *Asphalt* class, their outputs remain infiltrated by internal static and scattered edge noise points. CEGCN visibly breaks down when confronted with irregular geometric bends, misjudging the intersections between commercial buildings and road networks. Driven by blocking effects, SACNN forcibly merges small-scale details in a way that imposes a catastrophic loss of visual granularity. Post-denoising via SSLSM still leaves jagged “burrs” along inter-class borders. Likewise, 3DCT fails to completely suppress isolated variegated clustering in highly localized urban pockets. MSFI-CNet can effectively capture the high-frequency texture details of artificial materials, but encounters difficulty when handling the spectral heterogeneity and intra-class variation of natural surface categories. SSFEN almost perfectly recognizes artificial features such as the *Painted Metal Sheets* and *Asphalt* classes, which have stable spectra and clear boundaries; however, its accuracy drops sharply on categories consisting of mixed objects, such as *Gravel* and *Bare Soil*, exposing the shortcomings of spatial context modeling in cases requiring adaptation to complex objects. Conversely, the classification map of the proposed algorithm visualizes *Bare Soil* as exceptionally pure, traces *Asphalt* vectors with razor-sharp continuity, distinctively isolates *Meadows* from surrounding structures, and perfectly resolves scattered fine-grained objects. It comprehensively surpasses alternative architectures concerning geometric boundary integrity, detail retention, and ambient noise elimination.

**Figure 10:**
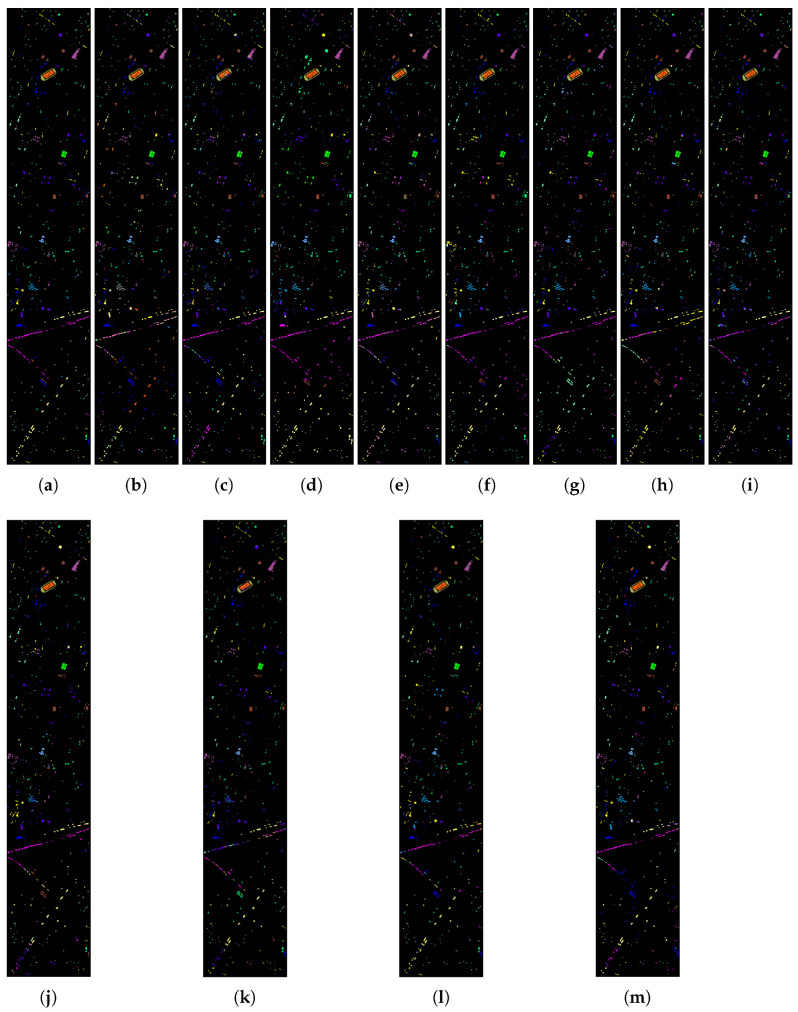
Classification maps generated by different methods on the Houston 2013 dataset: (**a**) ground truth, (**b**) SVM, (**c**) RPNet, (**d**) DIKS, (**e**) GRR, (**f**) M3FUNET, (**g**) CEGCN, (**h**) SACNN, (**i**) SSLSM, (**j**) 3DCT, (**k**)MSFI-CNet, (**l**) SSEFN, and (**m**) proposed method.

Finally, the proposed network sustains its preeminence across all relevant metrics on the HOU dataset. Interestingly, as shown in the Table 8, DIKS logs the lowest comparative classification accuracy on the HOU dataset. When DIKS engages the fine-grained and large-scale spatial footprints characteristic of the HOU dataset, it falls victim to a stark contradiction: aggressively limiting the superpixel count injects critical spatial noise, whereas maximizing it triggers devastating feature redundancy. SVM marginally outperforms DIKS, while RPNet and its direct descendants (GRR, M3FUNET) produce OA scores clustered between 66% and 70%. The OA value of MSFI-CNet reaches 72.81%. The four fully learnable deep architectures (CEGCN, SACNN, SSLSM, and 3DCT) all produce close results, ranging from 76% to 78%. The proposed algorithm again delivers dominant performance, achieving an OA margin 0.99% higher than its closest peer. As shown in the Figure 10, when visually analyzing the HOU classification mappings, SVM and DIKS produce results that are obfuscated by dense speckled noise fields. Although the optimized architectures of RPNet, GRR, and M3FUNET are able to suppress this noise, line continuity (e.g., roads and tracks) is frequently fractured. CEGCN fractures severely along irregular geographic boundaries, repeatedly failing to resolve complex junctions between disparate ground classes. SACNN again forcibly absorbs fine-grained topological details, destroying line signatures via strict superpixel amalgamation. SSLSM suffers from persistent border artifacts and insufficient spatial structure conformity. Although 3DCT advantageously leverages global representations, it also allows isolated misclassifications in specialized subregions. MSFI-CNet excels at capturing spatial patterns of rule-based artificial structures, but severely lacks the ability to identify subtle differences in natural vegetation spectra. SSFEN is stable in classifying categories with unique spectra and regular structures, such as *Synthetic grass*, *Water*, and *Tennis Court*; however, artificial building/road categories such as *Parking Lot 2*, *Parking Lot 1*, and *Road* have significantly lower accuracy due to high spectral similarity and broken edges, indicating that the ability of spatial spectral fusion to distinguish subtle differences needs to be improved. Exceeding all these paradigms, the classification layout rendered by our proposed model represents a definitive leap forward in resolving target boundaries, identifying fine-grained spatial variations, and rigorously suppressing classification noise.

**Figure 11:**
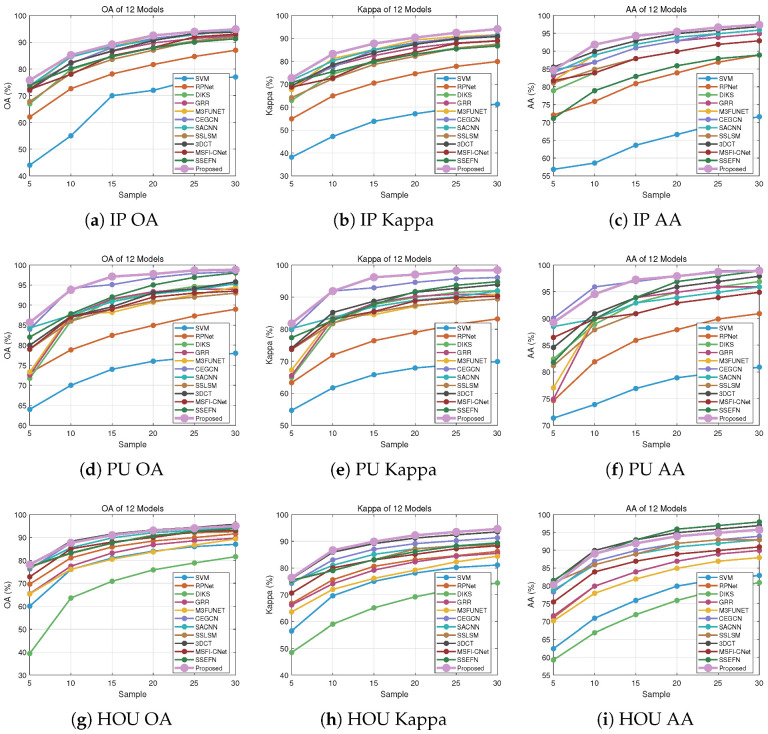
Classification accuracy corresponding to different training sample sizes across the three HSI datasets.

Lastly, we evaluate the classification performance of the proposed model and comparative methods with different training sample sizes. As shown in Figure 11, increasing the number of training samples produces high OA, Kappa, and AA scores for all of the models. The proposed model shows superior results compared to the others, especially in cases with limited training data, which further validates the effectiveness of the proposed model.

To verify the complexity of the proposed model, we choose the execution time from feature extraction to final classification as the performance evaluation criterion, since the training-free models do not have a training process. Table 9 shows the execution times of all the methods. It can be seen that the proposed model is superior in terms of efficiency, especially on the Pavia University dataset, where it consumes relatively less time while achieving good results. On the Houston 2013 dataset, more time is spent on superpixel segmentation due to the need to segment extremely fine superpixels; however, it still achieves better OA results with less time consumption compared to DIKS.

## 5. Conclusions

In this paper, we propose a novel training-free deep learning model for effective HSI classification, which we term Superpixel Random Selection Random Walk Multi-Branch Depthwise Convolutional Neural Network (SRSRWMD-CNN). First, various superpixel groups are generated through a model structure consisting of multi-scale superpixel segmentation and inter-layer multi-scale intra-layer grouping. We randomly select a predetermined number of superpixels from these groups as untrained convolution kernels, allowing the model to perceive features at different scales while capturing both local details and global contextual information. In addition, the integration of multi-branch deep convolution effectively alleviates the error accumulation in the process of multidimensional feature fusion while greatly enriching the spatial spectral representation. Meanwhile, the proposed superpixel random walk kernel generation strategy enhances the robustness of convolution, providing a significantly wider receptive field compared to existing deep neural networks that are limited by predefined rule-based convolution architectures.

However, the proposed model still has some limitations for. First, although the training-free framework utilizes convolutional kernels sampled from the dataset, it has a number of methodological limitations. The convolutional kernel is obtained from the source training data and its features are strongly correlated with the original data, which raises doubts about the performance in cross-domain tasks. In addition, the kernel generation process in our proposed framework involves two independent sources of randomness, namely, random selection of superpixels and random walks of superpixel convolution kernels. This dual randomness amplifies the differences in model performance.

Building on this work, future research seeking ways to effectively extract both local and global spatial–spectral features within the framework of training-free deep learning models could investigate superpixel-based training-free CNNs, GCNs, and fused models.

## Figures and Tables

**Table 1:** Details of the Indian Pines dataset (background category excluded).

#	Category	Train	Test	Samples/Pixels
1	Alfalfa	5	41	46
2	Corn-notill	5	1423	1428
3	Corn-mintill	5	825	830
4	Corn	5	232	237
5	Grass-pasture	5	478	483
6	Grass-tree	5	725	730
7	Grass-pasture-mowed	5	23	28
8	Hay-windrowed	5	473	478
9	Oats	5	15	20
10	Soybean-notill	5	967	972
11	Soybean-mintill	5	2450	2455
12	Soybean-clean	5	588	593
13	Wheat	5	200	205
14	Woods	5	1260	1265
15	Buildings-Tree-Drives	5	381	386
16	Stone-Steel-Towers	5	88	93
Total		80	10,169	10,249

**Table 2:** Details of the Pavia University dataset (background category excluded).

#	Category	Train	Test	Samples/Pixels
1	Asphalt	5	6626	6631
2	Meadows	5	18,644	18,649
3	Gravel	5	2094	2099
4	Trees	5	3059	3064
5	Painted Metal Sheets	5	1340	1345
6	Bare Soil	5	5024	5029
7	Bitumen	5	1325	1330
8	Self-blocking Bricks	5	3677	3682
9	Shadows	5	942	947
Total		45	42,731	42,776

**Table 3:** Details of the Houston 2013 dataset (background category excluded).

#	Category	Train	Test	Samples/Pixels
1	Healthy grass	5	1246	1251
2	Stressed grass	5	1249	1254
3	Synthetic grass	5	692	697
4	Trees	5	1239	1244
5	Soil	5	1237	1242
6	Water	5	320	325
7	Residential	5	1263	1268
8	Commercial	5	1239	1244
9	Road	5	1247	1252
10	Highway	5	1222	1227
11	Railway	5	1230	1235
12	Parking Lot 1	5	1228	1233
13	Parking Lot 2	5	464	469
14	Tennis Court	5	423	428
15	Running Track	5	655	660
Total		75	14,954	15,029

**Table 4:** Ablation experiment results on the three datasets.

Res2Net	Multiscale	Grouping	Random Selection	Depthwise Convolution	Multi-Branch	Superpixel Random Walk Kernel Generation	IP		PU		HOU	
							OA	Kappa	OA	Kappa	OA	Kappa
	✓	✓	✓	✓	✓	✓	73.98	70.70	79.87	74.61	70.19	67.77
✓		✓	✓	✓	✓	✓	74.61	71.42	83.23	78.77	72.51	70.29
✓	✓		✓	✓	✓	✓	74.84	71.69	79.04	73.40	75.41	73.41
✓	✓	✓		✓	✓	✓	74.84	71.69	85.08	81.00	65.10	62.29
✓	✓	✓	✓		✓	✓	66.37	62.07	67.29	59.54	73.02	73.02
✓	✓	✓	✓	✓		✓	68.71	64.68	69.04	62.18	75.41	73.41
✓	✓	✓	✓	✓	✓		67.56	63.55	81.36	76.39	77.76	75.96
✓	✓	✓	✓	✓	✓	✓	75.78	72.68	85.17	81.25	78.78	77.06

**Table 5:** Comparison of classification accuracy between superpixel dilated kernel generation, superpixel random shape kernel generation, and superpixel random walk kernel generation.

Superpixel Dilated Kernel Generation	Superpixel Random Shape Kernel Generation	Superpixel Random Walk Kernel Generation	IP		PU		HOU	
			OA	Kappa	OA	Kappa	OA	Kappa
✓			73.96	70.80	83.22	78.44	77.15	75.29
	✓		74.33	71.06	84.31	79.88	77.21	76.21
✓	✓		74.57	71.35	83.88	79.32	76.50	74.59
		✓	75.78	72.68	85.17	81.25	78.78	77.06

**Table 6:** Classification accuracy (%) AND Kappa coefficients for the IP dataset with five labeled samples per class. Underlined bold values indicate the optimal results.

Class	SVM	RPNet	DIKS	GRR	M3FUNET	CEGCN	SACNN	SSLSM	3DCT	MSFI-CNet	SSEFN	Proposed
Alfalfa	83.66	59.57	98.78	99.76	**100.00**	98.10	99.02	99.51	95.34	99.76	48.13	99.27
Corn-notill	27.75	54.89	34.06	56.58	**69.74**	58.49	68.59	49.24	52.65	63.38	66.88	67.41
Corn-mintill	25.31	**72.07**	48.82	63.39	59.54	61.77	48.74	61.45	66.58	58.59	60.52	52.36
Corn	49.31	57.54	78.79	91.16	82.41	97.60	91.98	92.24	40.28	84.14	49.87	**94.83**
Grass-pasture	57.53	69.83	81.59	80.04	75.44	80.90	77.07	72.28	89.10	77.57	**94.31**	85.82
Grass-tree	66.83	72.57	82.70	89.71	76.44	86.97	87.85	83.13	96.22	83.82	**96.79**	86.72
Grass-pasture-mowed	91.74	93.48	92.61	99.57	97.39	**100.00**	98.26	**100.00**	56.09	98.26	42.67	98.26
Hay-windrowed	66.43	87.42	96.03	99.05	87.32	99.81	95.10	95.33	**100.00**	87.17	97.78	95.56
Oats	75.33	99.33	93.33	**100.00**	**100.00**	**100.00**	98.67	**100.00**	42.85	98.67	43.07	**100.00**
Soybean-notill	45.02	58.12	50.78	69.05	65.19	**75.39**	68.95	73.82	78.37	69.11	62.15	73.97
Soybean-mintill	38.77	49.98	59.44	57.96	58.44	63.35	67.18	52.01	69.75	63.73	**82.44**	67.66
Soybean-clean	18.98	56.60	74.56	65.37	74.71	62.02	**74.76**	57.43	65.42	70.36	55.91	74.03
Wheat	86.70	97.90	95.35	98.75	91.75	**99.16**	89.65	96.25	81.48	85.90	87.51	89.25
Woods	67.29	70.79	**96.24**	86.06	84.70	86.41	90.52	83.97	88.72	85.84	95.71	89.82
Buildings-Tree-Drives	22.68	69.58	**92.39**	85.28	87.82	85.56	87.85	88.48	58.65	86.35	85.08	84.93
Stone-Steel-Towers	86.02	78.64	87.05	85.11	96.82	**100.00**	98.64	92.16	75.21	95.00	68.91	95.23
OA	44.63	59.57	66.94	71.60	71.31	73.58	74.91	67.80	72.43	72.36	74.00	**75.78**
Kappa	38.17	54.89	62.93	68.15	67.63	70.49	71.73	64.16	69.27	68.75	70.78	72.68
AA	56.83	72.07	78.91	83.09	81.72	84.72	83.93	81.08	**85.52**	81.72	71.11	84.69

**Table 7:** Classification accuracy (%) AND Kappa coefficients for the PU dataset with five labeled samples per class. Underlined bold values indicate the optimal results.

Class	SVM	RPNet	DIKS	GRR	M3FUNET	CEGCN	SACNN	SSLSM	3DCT	MSFI-CNet	SSEFN	Proposed
Asphalt	58.48	71.29	60.38	71.68	67.48	88.70	82.86	48.18	81.19	82.68	**97.47**	85.78
Meadows	65.47	71.61	62.47	70.47	66.86	74.79	78.97	84.38	86.40	70.97	**96.35**	79.42
Gravel	40.74	54.99	84.85	69.64	82.47	**89.97**	89.68	83.20	73.03	81.51	61.11	89.58
Trees	86.24	82.84	69.04	55.44	62.90	83.00	85.51	71.94	67.30	**87.15**	73.68	86.62
Painted M	98.87	99.43	97.72	97.20	96.10	99.84	98.04	**100.00**	99.25	98.44	99.65	98.01
Bare Soil	40.74	58.30	93.33	86.67	92.69	**96.14**	95.51	92.81	74.44	79.90	68.62	94.20
Bitumen	89.90	75.99	**99.40**	82.37	99.09	99.27	99.26	89.70	86.03	96.24	73.24	99.34
Self-blocking	61.91	71.53	78.86	67.04	75.44	**90.67**	84.66	86.84	65.31	89.32	74.44	87.84
Shadows	**99.86**	85.68	95.80	73.65	48.39	87.97	81.92	73.46	67.52	91.89	91.60	83.33
OA	64.01	71.30	71.74	72.43	73.52	83.99	84.29	79.44	79.90	79.19	82.02	**85.17**
Kappa	54.68	63.31	64.93	65.49	67.25	79.83	80.19	73.66	74.06	73.71	77.36	**81.25**
AA	71.36	74.63	82.43	74.91	77.03	**90.04**	88.49	81.17	84.57	86.45	81.8	89.35

**Table 8:** Classification accuracy (%) AND Kappa coefficients for the HOU dataset with five labeled samples per class. Underlined bold values indicate the optimal results.

Class	SVM	RPNet	DIKS	GRR	M3FUNET	CEGCN	SACNN	SSLSM	3DCT	MSFI-CNet	SSEFN	Proposed
Healthy grass	75.03	79.85	53.83	79.94	54.57	85.70	77.03	**91.34**	86.29	63.42	87.83	79.46
Stressed grass	84.28	71.56	25.00	74.85	61.41	60.21	80.31	76.73	83.67	71.19	**88.78**	82.63
Synthetic grass	98.01	91.89	**99.88**	99.93	91.47	99.92	98.21	97.64	99.18	95.45	99.54	97.21
Trees	**89.90**	64.72	35.84	75.68	49.47	87.84	81.95	72.97	89.00	67.46	85.12	78.75
Soil	90.44	72.89	65.70	89.39	49.47	94.85	89.17	**100.00**	99.98	94.29	88.06	96.23
Water	83.28	80.25	82.94	80.16	79.00	88.99	82.81	97.03	**99.08**	78.63	96.51	91.03
Residential	39.60	**78.24**	34.47	63.13	51.77	75.29	68.27	68.58	68.77	56.69	77.24	69.66
Commercial	32.45	41.98	14.46	42.15	39.85	35.56	64.62	52.03	51.28	60.25	**72.16**	62.87
Road	69.46	69.88	18.23	48.81	50.19	72.77	72.52	67.98	51.23	72.49	68.45	**73.30**
Highway	9.16	75.06	**76.86**	61.54	75.10	76.61	60.53	80.61	68.93	69.76	68.85	76.28
Railway	50.97	58.11	82.82	67.04	**85.15**	77.89	63.02	62.32	69.54	68.57	71.56	69.84
Parking Lot 1	12.13	56.26	33.18	45.72	67.48	67.52	69.85	57.43	70.01	70.62	65.56	**78.10**
Parking Lot 2	9.46	59.74	65.95	51.88	71.19	51.03	72.07	88.36	**90.17**	71.25	63.07	78.10
Tennis Court	93.36	85.22	**100.00**	96.34	99.91	99.98	94.59	**100.00**	99.81	95.91	96.82	98.96
Running Track	98.89	80.35	99.77	97.07	92.03	**100.00**	98.38	99.97	99.97	95.98	92.78	95.13
OA	59.57	69.22	52.07	68.69	66.25	76.20	76.52	77.08	77.79	72.81	77.25	**78.78**
Kappa	56.46	66.76	48.46	66.18	63.55	74.25	74.61	75.26	76.02	70.60	75.42	**77.06**
AA	62.43	71.07	59.26	71.57	70.17	78.27	78.76	80.86	81.25	75.46	**81.49**	80.81

**Table 9:** Execution time of different methods (in seconds).

Dataset	SVM	RPNet	DIKS	GRR	M3FUNET	CEGCN	SACNN	SSLSM	3DCT	MSFI-CNet	SSEFN	Proposed
Indian Pines	0.58	0.72	6.07	3.01	43.29	9.06	11.07	770.00	729.00	10.87	8.6	28.18
Pavia University	1.20	2.27	64.90	19.56	58.03	116.00	140.30	3071.00	2664.00	133.4	120.30	28.67
Houston 2013	1.85	6.11	7704.00	226.26	385.30	346.00	416.20	3111.00	1091.00	449.80	348.80	1034.00

## Data Availability

The original data presented in the study are openly available in Hyperspectral Remote Sensing Scenes at https://alweb.ehu.es/ccwintco/index.php?title=Hyperspectral_Remote_Sensing_Scenes (accessed on 24 May 2026).
